# The 12-item medical outcomes study short form health survey version 2.0 (SF-12v2): a population-based validation study from Tehran, Iran

**DOI:** 10.1186/1477-7525-9-12

**Published:** 2011-03-07

**Authors:** Ali Montazeri, Mariam Vahdaninia, Sayed Javad Mousavi, Mohsen Asadi-Lari, Sepideh Omidvari, Mahmoud Tavousi

**Affiliations:** 1 Department of Mental Health, Iranian Institute for Health Sciences Research, ACECR, Tehran, Iran; 2 Department of Social Medicine, Iranian Institute for Health Sciences Research, ACECR, Tehran, Iran; 3 Department of Physical Therapy, Faculty of Rehabilitation Sciences, Tehran University of Medical Sciences, Tehran, Iran; 4 Department of Epidemiology, Tehran University of Medical Sciences, Tehran, Iran; 5 Department of Family Health, Iranian Institute for Health Sciences Research, ACECR, Tehran, Iran

## Abstract

**Background:**

The SF-12v2 is the improved version of the SF-12v1. This study aimed to validate the SF-12v2 in Iran.

**Methods:**

A random sample of the general population aged 18 years and over living in Tehran, Iran completed the instrument. Reliability was estimated using internal consistency and validity was assessed using known-groups comparison and convergent validity. In addition the factor structure of the questionnaire was extracted by performing both exploratory and confirmatory factor analyses (EFA and CFA).

**Results:**

In all, 3685 individuals were studied (1887male and 1798 female). Internal consistency for both summary measures was satisfactory. Cronbach's α for the Physical Component Summary (PCS-12) was 0.87 and for the Mental Component Summary (MCS-12) it was 0.82. Known-groups comparison showed that the SF-12v2 discriminated well between men and women and those who differed in age and educational status (P < 0.05). Furthermore, as hypothesized the physical functioning, role physical, bodily pain and general health subscales correlated higher with the PCS-12, while the vitality, social functioning, role emotional and mental health subscales correlated higher with the MCS-12. Finally the exploratory factor analysis indicated a two-factor structure (physical and mental health) that jointly accounted for 59.9% of the variance. The confirmatory factory analysis also indicated a good fit to the data for the two-latent structure (physical and mental health).

**Conclusion:**

Although the findings could not be generalized to the Iranian population, overall the findings suggest that the SF-12v2 is a reliable and valid measure of health related quality of life among Iranians and now could be used in future health outcome studies. However, further studies are recommended to establish its stability, responsiveness to change, and concurrent validity for this health survey in Iran.

## Background

The SF-12 is the abridged practical version of the 36-item Short Form Health Survey (SF-36) that is developed as an applicable instrument for measuring health-related quality of life [1,2]. The instrument contains eight subscales as original 36-item questionnaire: physical functioning (PF, 2 items), role limitations due to physical problems (RP, 2 items), bodily pain (BP, 1 item), general health perceptions (GH, 1 item), vitality (VT, 1 item), social functioning (SF, 1 item), role limitations due to emotional problems (RE, 2 items) and mental health (MH, 2 items). The psychometric properties and factor structure of the SF-12 have been examined in several studies worldwide. Overall all results have indicated that the instrument is a reliable and valid measure that can be used in a variety of population groups [3-9].

The SF-12v2 has yielded a number of changes from Version 1 including item wording and response options. The response options have been extended for items of the RP and RE scales from 2 to 5 whilst the response categories for VT and MH items have been reduced from 6 to 5. Moreover two items are reworded [10]. Although the SF-12version 2 gives estimates of all 8 domains, there is more interest to focus on two distinct overall physical and mental health concepts known as Physical Component Summary (PCS) and Mental Component Summary (MCS).

The reliability and validity of the SF-12v2 has been investigated in numerous studies. The results of Medical Expenditure Panel Survey (MEPS) has shown that both component scores of the SF-12v2 have adequate reliability and validity and should be suitable for use in a variety of proposes within this database [11]. The Chinese version of the instrument has also acknowledged as an appropriate health indicator in Chinese adolescents [12]. In addition it has been demonstrated that the measure is suitable for assessment of health status in a variety of population groups such as diabetes [13], rheumatoid arthritis [14], hemophilia [15], cervical and lumbosacral disorders [16] and other health-related conditions [17-20].

Although in recent years we were witnessed the development of several health-related quality of life instruments in Iran [see http://www.Qolbank.ir], the Iranian versions of the well-developed, and well-known questionnaires still are lacking. Since 1997 we are working with Medical Outcome Trust and now QualityMetric Inc. to provide Iranian standard versions for one of the most popular general health-related quality of life instruments that is the Short Form Health Survey. It was hoped this might contribute to the existing literature and help both researchers and health professionals to have an opportunity to use the questionnaire in their potential research and practices. Thus, as part of a large study on the application of urban health equity assessment and response tool (Urban HEART) in Tehran [21], and alongside with our previous efforts [22,23], the aim of this study was to investigate the psychometric properties of the Iranian version of SF-12v2 among a general Iranian population. The second objective of the study was to establish normative data for the questionnaire in Iran.

## Methods

### The questionnaire and scoring

Permission was asked from the QualityMetric Inc. to develop the Iranian version of SF-12v2 (License agreement #CT103890/OP008065). Since we have previously developed the Iranian version of the SF-36v1 and SF-12v1 [22,23], the SF-12v2 was provided from the SF-12v1 and was used in this study.

To calculate the PCS-12 and the MCS-12 scores we used the QualityMetric Health Outcomes Scoring Software 2. The software uses all the 12 items to produce scores for the PCS-12 and the MCS-12 and applies a norm-based scoring algorithm empirically derived from the data of a US general population survey [24]. It has been recommended that the US-derived summary scores, that assume a mean of 50 and a standard deviation (SD) of 10, be used in order to facilitate cross-cultural comparison of results [2,4]. In theory the possible scores for the PCS-12 and the MCS-12 could be ranged from 0 (the worst) to 100 (the best).

### Data collection

A cross-sectional population-based study was conducted in Tehran, Iran in 2009. The ethics committee of the Iranian Center for Education, Culture and Research (ACECR) approved the study. The Iranian version of SF-12v2 was administered to a random sample of individuals aged 18 years and over. To select a representative sample of the general population a multi-stage area sampling procedure was applied. Every household within 22 municipal districts in Tehran had the same probability to be sampled. A team of trained interviewers collected data and all participants were interviewed in their home. The interviews were carried out with individual's informed consent.

### Statistical analysis

In addition to descriptive statistics (including floor and ceiling effects), according to International Quality of Life Assessment (IQOLA) Project to assess the psychometric properties of the Iranian version of SF-12v2 several tests were performed. To test reliability, the internal consistency for summary measures was estimated using Cronbach's alpha coefficient and alpha equal to or greater than 0.70 was considered satisfactory [25]. Validity was assessed using known-groups comparison to test how well the instrument discriminates between subgroups of the study sample that differed in their health conditions. This was a separate item in the introductory part of the questionnaire asking each respondent to report if they were suffering from a chronic illness. This included recording of cardiovascular, musculoskeletal, gastrointestinal, hematological, neurological and chronic respiratory diseases, diabetes, and cancers. It was expected that those who reported to be free of a chronic condition would have higher scores in all measures than those who reported to have one or more chronic conditions [1]. The t-test was used for comparison. Furthermore convergent validity was assessed performing item-scale correlations. This approach is to examine the correlation between similar attributes as to establish convergent validity (known as multitrait analysis) [26]. Correlations were calculated using Spearman's correlation coefficient (rho). It was expected that item scores would correlate higher with own hypothesized scale than other scales and PF, RP, BP and GH scores would correlate higher with the PCS-12 whether the VI, SF, RE and MH scores would correlate higher with the MCS-12. Correlation values of 0.40 or above were considered satisfactory (r ≥ 0.81-1.0 as excellent, 0.61-0.80 very good, 0.41-0.60 good, 0.21-0.40 fair and 0.20 poor) [25].

The factor structure of the questionnaire was extracted by performing both exploratory factor analysis (EFA) and confirmatory factor analysis (CFA). Exploratory factor analysis was performed using the principal component analysis with obligue rotation. It was hypothesized that a two-factor solution would be obtained with eigenvalues greater than 1. Finally, confirmatory factor analysis was performed while a two-factor model (physical component summary and mental component summary) was specified for the analysis. We report several goodness-of-fit indicators including: goodness of fit index (GFI), adjusted goodness of fit index (AGFI), the root mean square error of approximation (RMSEA), normed fit index (NFI), and comparative fit index (CFI). The GFI and AGFI are chi-square based calculations independent of degrees of freedom. The recommended cut-off values for acceptable values are ≥ 0.90. The RMSEA tests the fit of the model to the covariance matrix. As a guideline, values of < 0.05 indicate a close fit and values below 0.11 are an acceptable fit. The NFI and CFI values range from 0 to 1 with a value of greater than 0.90 being acceptable fit to the data [27,28].

## Results

In all 4337 individuals were approached. Of these, 3685 individuals (1887 male and 1798 female) agreed to take part in the study, giving a response rate of 85.0%. The mean age of the respondents was 35.6 (SD = 14.7) and mostly had secondary education (51.1%). The demographic characteristics of the study sample are shown in Table 1.

**Table 1:** Demographic characteristics of the study sample (n = 3685)

		Number (%)
**Age groups (year)**		

	18-24	832 (22.6)

	25-34	369 (10.0)

	35-44	654 (17.7)

	45-54	912 (24.7)

	55-64	786 (21.4)

	≥ 65	132 (3.6)

	Mean (SD)	35.6 (14.7)

**Gender**		

	Male	1887(51.0)

	Female	1798(49.0)

**Marital status**		

	Single	1039(28.2)

	Married	2011(54.5)

	Widowed/divorced	635(17.3)

**Educational status**		

	Primary	895 (24.3)

	Secondary	1882 (51.1)

	Higher	908 (24.6)

**Employment status**		

	Employed	1622 (44.0)

	Housewife	888 (24.1)

	Student	796 (21.6)

	Unemployed	182 (5.0)

	Retired	197 (5.3)

The results showed that both summary measures exceeded the 0.70 level for Cronbach's alpha indicating satisfactory results (α for the PCS-12 and the MCS-12 was 0.87 and 0.82 respectively). The mean score for the PCS-12 was 42.3 (SD = 11.4) and for the MCS-12 it was 44.6 (SD = 11.9). For both the PCS-12 and the MCS-12 the percentage of respondents scoring at the lowest level (i.e. floor effect) and at the highest level (i.e. ceiling effect) was almost nothing (frequency was 1 for each). The descriptive statistics for the SF-12v2 scales and its summary measures are shown in Table 2. In addition to provide normative data for subgroups of the study sample the summary scores for different age groups, males and females and people with different level of education are presented in Table 3.

**Table 2:** Item description and descriptive statistics for the SF-12v2 component summary scores (n = 3685)

SF-12v2 item (scale)	Mean row scores (SD)	95% CI	Response frequencies (%)				
			**1**	**2**	**3**	**4**	**5**

Limitations in moderate physical activities (PF)	2.33 (0.76)	2.31-2.36	18.2	30.4	51.3	-	-

Limitations in climbing several flights of stairs (PF)	2.18 (0.80)	2.15-2.20	24.9	32.6	42.4	-	-

Accomplished less due to physical health (RP)	3.41 (1.29)	3.37-3.45	8.4	19.0	23.6	21.3	27.7

Limited in kind of work or activities due to physical health (RP)	3.55 (1.26)	3.51-3.59	6.8	15.5	25.2	21.1	31.4

Pain interference with work inside or outside home (BP)**	2.53 (1.15)	2.49-2.56	23.1	27.5	26.9	18.5	4.0

Health rating in general (GH)**	3.34 (1.01)	3.31-3.38	6.2	10.8	36.7	35.4	11.0

Interference of physical health or emotional problems with social activities (SF)	3.50 (1.19)	3.46-3.54	5.8	15.6	27.5	25.0	26.1

Accomplished less due to emotional problems (RE)	3.53 (1.26)	3.49-3.57	6.8	16.8	23.2	23.2	30.0

Not careful in work or activities due to emotional problems (RE)	3.62 (1.19)	3.58-3.65	5.0	14.5	24.9	25.2	30.4

Having a lot of energy (VT)**	2.86 (1.19)	2.83-2.90	15.0	25.0	27.9	22.7	9.4

Feel calm and peaceful (MH)**	2.49 (1.21)	2.45-2.53	24.3	31.5	22.9	13.6	7.7

Feel downhearted and blue (MH)	3.48 (1.27)	3.44-3.52	8.5	16.0	21.5	27.1	26.9

**Summary components**	**PCS**	**MCS**					

Mean (SD)***	42.3 (11.4)	44.6 (11.9)					

95% CI	41.9-42.6	44.2-45.0					

Cronbach's α	0.87	0.82					

Skewness	-0.40	-0.35					

Minimum (% floor)	4.70 (0.0)	5.88 (0.0)					

Maximum (%ceiling)	73.6 (0.0)	77.1 (0.0)					

* The format adapted from [4]

** Item recorded in order to make all response frequencies in the same direction. Now for all 12 items higher scores indicate better condition.

*** Derived form Quality Metric Health Outcomes Scoring Software 2.

**Table 3:** The SF-12v2 summary scores for the general population by gender, age, education, and chronic disease condition

	Physical component summary	Mental component summary
	**Mean (SD)**	**Mean (SD)**

**Age groups**		

18-24 (n = 832)	48.0 (6.7)	47.7 (13.5)

25-34 (n = 369)	47.5 (8.6)	46.1 (11.5)

35-44 (n = 654)	45.0 (9.4)	45.4 (12.0)

45-54 (n = 912)	45.0 (10.1)	44.1 (12.2)

55-64 (n = 786)	42.3 (11.6)	44.0 (12.0)

≥ 65 (n = 132)	35.5 (12.0)	43.4 (11.4)

P value**	< 0.001	0.03

**Gender**		

Male (n = 1887)	45.0 (10.0)	46.0 (11.7)

Female (n = 1798)	39.4 (12.0)	43.2 (12.0)

P value*	< 0.001	< 0.001

**Educational status**		

Primary (n = 895)	38.7 (12.0)	43.6 (11.6)

Secondary (n = 1882)	44.3 (10.1)	44.7 (12.2)

Higher (n = 908)	46.5 (10.2)	46.7 (11.5)

P value**	< 0.001	< 0.001

**Chronic disease**		

No (n = 3259)	43.4 (10.8)	45.9 (11.1)

Yes (n = 416)	33.4 (11.8)	34.3 (12.4)

P value*	< 0.001	< 0.001

**Chronic disease (older age groups only, n = 918)**		

No (n = 770)	37.0 (11.8)	45.5 (10.7)

Yes (148)	28.7 (10.0)	38.2 (12.5)

P value*	< 0.001	< 0.001

* Derived from t-test

** Derived from one-way analysis of variance (ANOVA)

Known-groups comparison showed that the SF-12v2 discriminated well between subgroups of people who were differed in their health condition. As hypothesized those without any chronic conditions scored higher on the PCS-12 and the MCS-12 than those with a chronic condition. To avoid the danger of colinearity between chronic pathology and age the same analysis was applied to older age groups only and the same results were obtained as expected (Table 3).

The results from correlation analysis demonstrated that item scores correlated higher with own hypothesized scale than other scales and that the PF, RP, BP, and GH subscales correlated higher with the PCS-12 score, while the VT, SF, RE, and MH subscales more correlated with the MCS-12 score lending support to its good convergent validity. Table 4 shows the results of item-scale correlation matrix for SF-12 subscales and summary measures.

**Table 4:** Item-scale correlation matrix for the eight SF-12v2 scales and summary measures*

	PF	RP	BP	GH	SF	RE	VT	MH	PCS	MCS
PF										

PF1	**0.93**	0.59	0.53	0.48	0.37	0.35	0.35	0.26	**0.80**	0.13

PF2	**0.94**	0.59	0.54	0.50	0.37	0.36	0.39	0.29	**0.81**	0.16

RP										

RP1	0.57	**0.94**	0.54	0.46	0.43	0.55	0.38	0.31	**0.69**	0.33

RP2	0.62	**0.94**	0.59	0.49	0.45	0.53	0.39	0.33	**0.74**	0.32

BP										

BP1	0.57	0.60	**1.00**	0.56	0.48	0.46	0.46	0.42	**0.75**	0.36

GH										

GH1	0.51	0.49	0.55	**0.98**	0.40	0.39	0.50	0.44	**0.66**	0.40

SF										

SF1	0.40	0.46	0.48	0.41	**1.00**	0.48	0.37	0.46	0.39	**0.63**

RE										

RE1	0.36	0.55	0.42	0.38	0.45	**0.94**	0.34	0.50	0.28.	**0.71**

RE2	0.35	0.53	0.44	0.38	0.46	**0.94**	0.35	0.49	0.27	**0.71**

VT										

VT1	0.39	0.41	0.46	0.50	0.37	0.37	**1.00**	0.49	0.43	**0.58**

MH										

MH1	0.24	0.28	0.37	0.41	0.37	0.39	0.51	**0.83**	0.16	**0.71**

MH2	0.25	0.30	0.34	0.35	0.43	0.50	0.33	**0.85**	0.11	**0.74**

* Figures are Spearman's correlation coefficient (rho). All correlations were significant at the 0.01 levels. Correlation values of 0.4 or above were considered satisfactory (correlations ≥ 0.81-1.0 as excellent, 0.61-0.80 very good, 0.41-0.60 good, 0.21-0.40 fair, and 0-0.20 poor) [25].

Principal component analysis with oblique rotation loaded two factors. The results are shown in Table 5. Eigenvalues for the two factors that explained most of the variance observed was 5.80 and 1.37 respectively. The two-factor structure (physical and mental health) jointly accounted for 59.9% of the variance. The results indicated that PF, RP, BP, and GH items loaded higher on the physical health component and VT, SF, RE, and MH loaded higher on the mental health component.

**Table 5:** Factor structure of the SF-12v2 derived from principal component analysis*

	Factor 1	Factor 2
**Physical functioning (PF)**		

Limitations in moderate physical activities (PF1)	**0.84**	0.31

Limitations in climbing several flights of stairs (PF2)	**0.85**	0.34

**Role physical (RP)**		

Accomplished less due to physical health (RP1)	**0.79**	0.51

Limited in kind of work or activities due to physical health (RP2)	**0. 83**	0.50

**Bodily pain (BP)**		

Pain interference with work inside or outside home (BP)**	**0.75**	0.56

**General health (GH)**		

Health rating in general (GH1)	**0.65**	0.55

**Social functioning (SF)**		

Interference of physical health or emotional problems with social activities (SF1)	0.27	**0.65**

**Role emotional (RE)**		

Accomplished less due to emotional problems (RE)	0.49	**0.78**

Not careful in work or activities due to emotional problems (RE)	0.48	**0.78**

**Vitality (VT)**		

Having a lot of energy (VT1)	0.50	**0.61**

**Mental health (MH)**		

Feel calm and peaceful (MH1)	0.29	**0.71**

Feel downhearted and blue (MH2)	0.27	**0.74**

**Eigenvalues**	*5.80*	*1.37*

**Variance explained (%)**	*48.4*	*11.5*

* Values equal or greater than 0.4 were considered satisfactory

**Figure 1:**
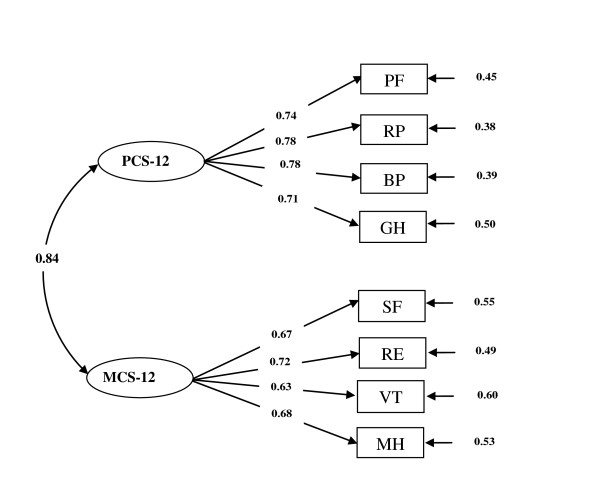
**A two-factor model for the SF-12v2 obtained from confirmatory factor analysis**.

Finally, the results for confirmatory factor analysis are shown in Figure 1. The two-factor model, that is physical component summary (PCS-12) and mental component summary (MCS-12), was specified and tested. The results provided a good fit to the data lending support to the original hypothesized structure of the questionnaire with GFI = 0.93, AGFI = 0.87, RMSE = 0.10, 90% CI RMSE = 0.10 to 0.11, NFI = 0.96, and CFI = 0.96.

## Discussion

This study reported the psychometric properties of the Iranian version of SF-12v2 among a general population in Tehran. The results indicated that the instrument is a reliable and valid measure that can be used in monitoring and measuring population health status. Since the present study used the norm-based scoring algorithms for calculating the PCS-12 and the MCS-12, the results from this study also can be used for cross-cultural health-related quality of life comparisons. The psychometric properties of the SF-12v2 in different cultures are also showed satisfactory results [12,13]. Indeed evidence suggests that the instrument is applicable among diverse population clusters and is appropriate as a health status measure in subgroups of a population [14-17]. The findings from this study indicated that women, older age groups and people with lower educational status had poorer health compared to men, the younger respondents and those with better educational status. The findings are consistent with results from other studies carried out in different settings [12-14,22]. In addition, known groups comparison indicated that the SF-12v2 summary components were able to distinguish very well between subgroups of the respondents who differed in chronic health problem.

This study used a relatively large sample of the general population. Therefore as it has been suggested [29] that the results of this study might be considered as Iranian normative data for the 12-item Short Form Health Survey version 2 (SF-12v2) and perhaps could be used as a basis for comparison with specific populations in the future studies. However one might argue that a sample from capital is not necessarily representative of the entire country. In general this is true but since Tehran has become a multicultural metropolitan area it has been suggested that a sample from the general population in Tehran could be regarded as a representative sample of the general population in Iran [22]. The migration rate from the entire country to Tehran (due to its apparent attractiveness, facilities for living and opportunities for jobs etc.) is very high and vibrant. Usually in a random sample of the general population in Tehran the possibility to reach people from almost all part of the Iran is very likely.

The hypothesis regarding the item component correlations also showed desirable results. As expected the PF, RP, BP and GH subscales correlated higher with the PCS-12 while the VT, SF, RE and MH more correlated with the MCS-12 score (Table 4). This finding is somewhat different from those reported by the Ware et al. where physical functioning, role physical and bodily pain correlated most highly with the PCS and mental health, role emotional and social functioning correlated most highly with the MCS; and vitality, general health and social functioning had a relatively high correlation with both components [1]. However, a number of studies have shown that vitality item has appeared to correlate higher with the PCS than with the MCS score [4]. It is argued this might be due to cultural differences among people from different countries or simply this might be occurred due to translation problems [22,30]. In addition, it has been reported that even translation of concepts such as social functioning could be difficult in some Asian cultures [31]. As Ware indicates the most important empirical point that should be noted is the fact that scales that load highest on the physical component are most responsive to treatment that change physical morbidity whereas scales loading highest on the mental component respond to drugs and therapies that target mental health [32].

In general, the psychometric tests of the Iranian version of SF-12v2 showed satisfactory results. Principal component analysis with oblique rotation supported a two-factor structure for the instrument that ensured the original conceptual model of the instrument [1,2]. A recent study on driving the SF-12v2 physical and mental health summary scores with different scoring algorithms suggested the summary scores were more consistent with changes in individual scales when the oblique rotation was performed. The authors, thus, concluded that oblique rotation would be more preferable when performing factor analysis for the SF-12v2 [33]. In addition, the results obtained from the confirmatory factor analysis indicated that the two-factor model fitted the data very well. A study in Chinese adolescents reported that a one-factor structure also showed a satisfactory fit in the CFA [12].

The findings from this study indicated that overall the Iranian version of SF-12v2 performed better than the Iranian version of the SF-12v1. The Chrobach's alpha for the PCS and the MCS version 1 were 0.73 and 0.72 while for version 2 these were 0.87 and 0.82, respectively. Similarly the results from EFA indicated that the two-factor structure for version 1 jointly accounted for 57.8% of the variance observed whereas this for version 2 was 59.9% [23].

Although this study did not provide evidence for test-retest reliability, responsiveness to change or other psychometric tests; the findings showed that the Iranian version of SF-12v2 is a reliable instrument for measuring health-related quality of life. The future studies could focus on other psychometric properties of the questionnaire and also on different applications of the instrument. In addition, since the study sample was from Tehran, for the certainty data from this sample should not be generalized to the whole Iranian population. In fact this is a major limitation.

## Conclusion

In general the findings suggest that the SF-12v2 is a reliable and valid measure of health-related quality of life among Iranian population and now could be used in future health outcome studies. However, further studies are recommended to establish stronger psychometric properties for this health survey in Iran.

## Abbreviations

SF-12v2: The 12-item Short Form Health Survey version 2; PF: Physical Functioning; RP: Role Physical; BP: Bodily Pain; GH: General Health; VT: Vitality; SF: Social Functioning; RE: Role Emotional; MH: Mental Health; IQOLA: International Quality of Life Assessment; PCS: Physical Component Summary; MCS: Mental Component Summary; EFA: exploratory factor analysis; CFA: confirmatory factor analysis

## Competing interests

The authors declare that they have no competing interests.

## Authors' contributions

AM was the main investigator, provided the questionnaire, carried out the analysis, and wrote the paper. MV contributed to the analysis and the writing process. MAL contributed to the data collection and the study management. SJM contributed to the study design, and analysis. SO contributed to the study design and drafting. MT contributed to the CFA analysis. All authors read and approved the manuscript.
